# Tri-State Medical Society of Alabama, Georgia and Tennessee

**Published:** 1893-10

**Authors:** 


### TRI-STATE MEDICAL SOCIETY OF ALABAMA, GEORGIA AND TENNESSEE.

The Fifth Annual Meeting of the Tri-State Medical Society, will be held in the Unitarian Church, Chattanooga, October 17th, 18th and 19th. The prospects are for a large attendance, and a very interesting meeting. All are invited to attend the meeting. A circular will soon be issued giving a list of papers. The following is an incomplete list:

"Membranous Croup, with Report of Cases Treated with Tracheotomy," R. N. Harbin, Calhoun, Ga.

"Intubation," Max Thorner, Cincinnati, O.

"Treatment of Stone in the Cystic Duct," W. E. B. Davis, Birmingham, Ala.

"Cholecystotomy," Paul F. Eve, Nashville, Tenn.

"Symptoms and Pathology of Fractures About the Elbow," J. B. Murfree, Murfreesboro, Tenn.

"Treatment and Prognosis of Fractures About the Elbow," Willis F. Westmoreland, Atlanta, Ga.

"Pathology of the Sequelæ of Purulent Inflammation of the Middle Ear," T. Hilliard Wood, Nashville, Tenn.

"Treatment of the Sequelæ of Purulent Inflammation of the Middle Ear," G. C. Savage, Nashville, Tenn.

"Action of the Galvanic Current on the Uterine Tissue," H. Berlin, Chattanooga, Tenn.

"The Significance of Albumen in the Urine of Pregnancy," E. T. Camp, Gadsden, Ala.

"Tuberculosis on the Cumberland Mountains," L. P. Barbour, Tracy City, Tenn.

"Symptoms and Treatment of Gastritis," G. T. Prince, Whiteside, Tenn.

"Treatment of Varicocele," J. W. Handly, Nashville, Tenn.

"Treatment of Pyrexia," J. A. Witherspoon, Columbia, Tenn.

"Fevers of Alabama," R. M. Cunningham, Ala.

"Serious and Watery Discharges During Gestation," J. R. Rathmell, Chattanooga, Tenn.

"Treatment of Ophthalmia Neonatorum," B. F. Travis, Chattanooga, Tenn.

"Naso-Pharyngeal Adenoids," E. L. Jones, Chattanooga, Tenn.

"Treatment of Puerperal Mastitis," J. W. Russey, Chattanooga, Tenn.

Frank Trester Smith, Secretary, Chattanooga, Tenn.

---

### ATLANTA POLYCLINIC.

THE first quarterly report of clinic work shows over 1,100 cases admitted, over 3,500 treatments, with above 100 operations, including removal superior maxilla, amputation thigh at upper third, coeliotomy for extra uterine pregnancy, operation for vesico-vaginal fistula, etc.

---

DR. R. H. Hutchings, of New York, was married to Miss Belle Compton, of Milledgeville, Ga., on September 6th.

---

DR. Hudson, of Virginia, has moved here and is associated with Dr. W. C. Janigin.

SHARP & DOHME, of Baltimore, Md., have removed their general business department to New York City, 41 John St. Their recently enlarged and completely equipped laboratories remain in Baltimore. This firm has been in business thirty years. As to their preparations, they need no praise from us, as the profession over the world knows of their high standard.

WE had pleasant calls, during the month, from the following doctors. We are always glad to see them: Drs. W. W. Stewart, Columbus; Henry R. Slack, LaGrange; J. B. Reynolds, Lumber City; Jeff Davis, Toccoa; D. C. Daves, Skemit; Thos. H. Lyons, Martin. Hope all the doctors will visit us whenever they are in the city.

---

THE next meeting of the Southern Surgical and Gynecological Association will be held in New Orleans on the 14th, 15th and 16th of November. Members of the medical profession are cordially invited to attend.

---

DR. M. H. Oliver, a prominent physician of Ennis, Texas, died on the 8th of September. He was at the time a resident of this city and Professor in the Atlanta Medical College.

---

DR. James E. Reeves, of Chattanooga, Tenn., is being sued for alleged defamatory statements of the Amick Cure. We will watch the suit with a good deal of interest.

---

DRS. A. W. Calhoun and W. S. Goldsmith, of this city, have just returned from the World's Fair.

---

WE wish to call our readers attention to the advertisement of Codliver Glycerine Co.

---

DR. H. J. Speer, of Saginaw, Mich., has located here.

